# *Salmonella* Populations inside Host Cells

**DOI:** 10.3389/fcimb.2017.00432

**Published:** 2017-10-04

**Authors:** Sónia Castanheira, Francisco García-del Portillo

**Affiliations:** Laboratory of Intracellular Bacterial Pathogens, Departamento de Biotecnología Microbiana, Centro Nacional de Biotecnología-Consejo Superior de Investigaciones Científicas (CNB-CSIC), Madrid, Spain

**Keywords:** *Salmonella*, intracellular, cytosol, vacuole, heterogeneity

## Abstract

Bacteria of the *Salmonella* genus cause diseases ranging from gastroenteritis to life-threatening typhoid fever and are among the most successful intracellular pathogens known. After the invasion of the eukaryotic cell, *Salmonella* exhibits contrasting lifestyles with different replication rates and subcellular locations. Although *Salmonella* hyper-replicates in the cytosol of certain host cell types, most invading bacteria remain within vacuoles in which the pathogen proliferates at moderate rates or persists in a dormant-like state. Remarkably, these cytosolic and intra-vacuolar intracellular lifestyles are not mutually exclusive and can co-exist in the same infected host cell. The mechanisms that direct the invading bacterium to follow the cytosolic or intra-vacuolar “pathway” remain poorly understood. *In vitro* studies show predominance of either the cytosolic or the intra-vacuolar population depending on the host cell type invaded by the pathogen. The host and pathogen factors controlling phagosomal membrane integrity and, as consequence, the egress into the cytosol, are intensively investigated. Other aspects of major interest are the host defenses that may affect differentially the cytosolic and intra-vacuolar populations and the strategies used by the pathogen to circumvent these attacks. Here, we summarize current knowledge about these *Salmonella* intracellular subpopulations and discuss how they emerge during the interaction of this pathogen with the eukaryotic cell.

## Introduction

Pioneer studies by Takeuchi and colleagues, dated on 1967 (Takeuchi, 1967; Takeuchi and Sprinz, 1967), opened a new era for the analysis of *Salmonella* pathogenesis. Using electron microscopy techniques, these authors provided the first evidence for an intracellular location of this pathogen when it invaded the intestinal epithelium of guinea pigs. The studies described that, as bacteria advanced into the intestinal epithelial cell, they “became membrane-enclosed” by a process like that seen in macrophages (Takeuchi, 1967; Takeuchi and Sprinz, 1967). Subsequent *in vitro* studies involving bacterial infection of cultured epithelial HeLa cells confirmed the presence of *Salmonella* inside membrane-bound vacuoles (Kihlstrom and Latkovic, 1978). This intra-vacuolar location was further corroborated in all cell lines and cell types tested, including polarized epithelial cells (Finlay and Falkow, 1989). Despite the widely-accepted classification of *Salmonella* as a pathogen residing within membrane-bound vacuoles, the last decade has accumulated evidence supporting transit to the cytosol of some bacteria from this intra-vacuolar population. This review focuses on the differentiation of these two *Salmonella* populations, cytosolic and intra-vacuolar, and how these lifestyles are regulated by host and pathogen factors.

## A retrospective view to the *Salmonella*-containing vacuole (SCV) and *Salmonella* intracellular populations

**Figure 1:**
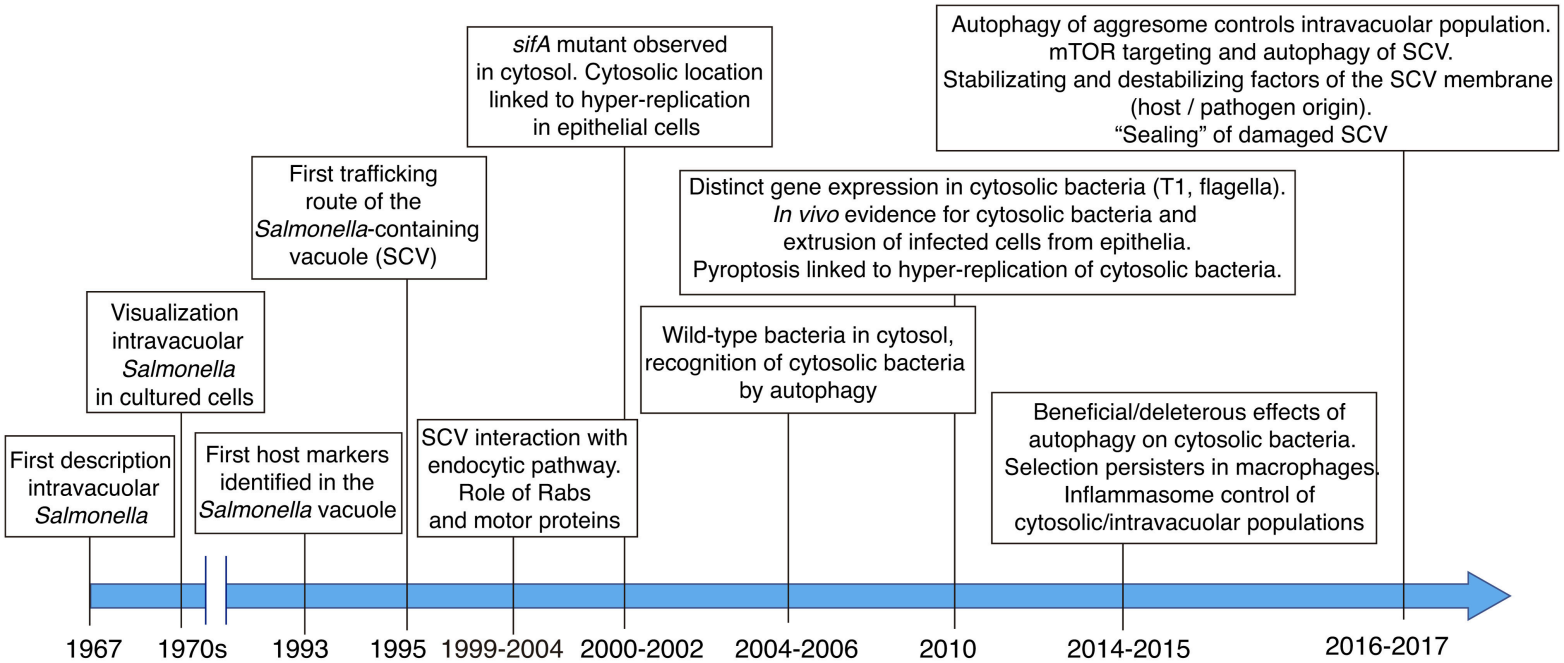
Main milestones in the discovery and subsequent characterization of the *Salmonella*-containing vacuole (SCV), the cytosolic population and the contribution of host and pathogen factors to the dynamics of the intra-vacuolar and cytosolic populations.

The *in vivo* studies by Takeuchi and colleagues and the analyses performed in the seventies focused mainly on tracking of the pathogen in infected cells using transmission electron microscopy (TEM). This technique allowed researchers to demonstrate the presence of a vacuolar membrane surrounding intracellular *Salmonella* (Figure 1). In the early nineties, studies based on immunofluorescence microscopy uncovered the identity of host proteins located in the vacuolar membrane surrounding intracellular *Salmonella*, specifically the lysosomal membrane glycoproteins Lamp-1 and Lamp-2 (García-del Portillo et al., 1993; García-del Portillo and Finlay, 1995; Figure 1). One of these studies coined the term *Salmonella*-containing vacuole (SCV) to this specialized phagosomal compartment (García-del Portillo and Finlay, 1995). Further studies focused on the analysis of other eukaryotic organelle markers in *Salmonella*-infected cells to dissect the trafficking route of the SCV (Martinez-Lorenzo et al., 2001; Catron et al., 2002; Steele-Mortimer et al., 2002). An important conclusion of these studies was that the SCV is a highly dynamic compartment undergoing loss and gain of Rab GTPases involved in early and intermediate steps of the endocytic pathway (Meresse et al., 1999; Steele-Mortimer et al., 1999; Brumell et al., 2001; Bakowski et al., 2010; Figure 1). Interaction of the cytoskeletal motor proteins kinesin and dynein with the SCV and the modulation of these interactions by effector proteins translocated by specialized type III secretion systems (T3SS), were also major breakthroughs in the field (Guignot et al., 2004; Harrison et al., 2004; Marsman et al., 2004; Boucrot et al., 2005; Dumont et al., 2007; Figure 1).

In a seminal paper, Holden and colleagues linked the function of SifA, an effector translocated by the T3SS encoded in *Salmonella* pathogenicity island 2 (SPI-2) -hereinafter referred as SPI2-T3SS-, to stability of the SCV membrane. These authors observed a high proportion of *Salmonella sifA* mutant bacteria “free” in the cytosol of epithelial cells (Beuzon et al., 2000). Further studies showed that some wild-type bacteria could also reach the cytosol and proliferate at higher rates than when they were intra-vacuolar (Brumell et al., 2002). Most of these initial studies were carried out in HeLa epithelial cells, therefore not providing clues about the vacuole-to-cytosol transition in other infection models. Moreover, although the macrophage and fibroblast cytosol were found not to be permissive for growth of the *sifA* mutant (Beuzon et al., 2002), a comparative study examining their bactericidal activity for wild-type *Salmonella* was not shown.

Later studies referred to a *Salmonella* population residing within injured SCV (Birmingham and Brumell, 2006). Damaged membranes expose glycans on their luminal side, which are decorated with ubiquitin and recognized by lectins like galectin-8. This glycan recognition is a danger signal that facilitates recruitment of autophagy proteins and, ultimately, autophagosome formation (Birmingham and Brumell, 2006; Thurston et al., 2012). How the distinct *Salmonella* intra-vacuolar subpopulations differing in SCV membrane integrity and the population of “free” cytosolic bacteria interconnect during the infection has not been yet examined in live-cells. Other studies showed that the damaged SCV can be “repaired,” either by increased recruitment of lysosomes with detrimental consequences for the pathogen (Roy et al., 2004) or, by the autophagy machinery allowing progression of the infection (Kreibich et al., 2015). Autophagy has also been proposed to promote replication of cytosolic bacteria (Yu et al., 2014). Considering these observations, it is probable that some intracellular *Salmonella* are only “transiently” exposed to the host cell cytosol. Autophagy can therefore have deleterious or beneficial effect to the pathogen depending on the host cell type or the infection time. More recent studies focused on the identification of host and pathogen factors that modulate integrity of the SCV membrane (see below).

### *Salmonella* intracellular populations in distinct host cell types

Several studies claim that a cytosolic population of wild-type *Salmonella* exists irrespectively of the host cell type examined (Perrin et al., 2004; Birmingham and Brumell, 2006; Birmingham et al., 2006; Meunier et al., 2014). Most of these studies however lack a temporal analysis of the cytosolic population to define whether it perpetuates along the infection or, by contrast, is transient and effectively eradicated by the host cell defenses. As an example, a recent work performed in rat and human fibroblast cell lines (NRK-49F and BJ-5ta) showed that intracellular bacteria are surrounded by a membrane containing lysosomal-membrane glycoproteins at all post-infection times (Lopez-Montero et al., 2016). This evidence discards the presence of a stable population of “free” cytosolic bacteria in this cell type. Here, we summarize these distinct features that vary depending the host cell type invaded by the pathogen.

### Epithelial cells and macrophages

**Figure 2:**
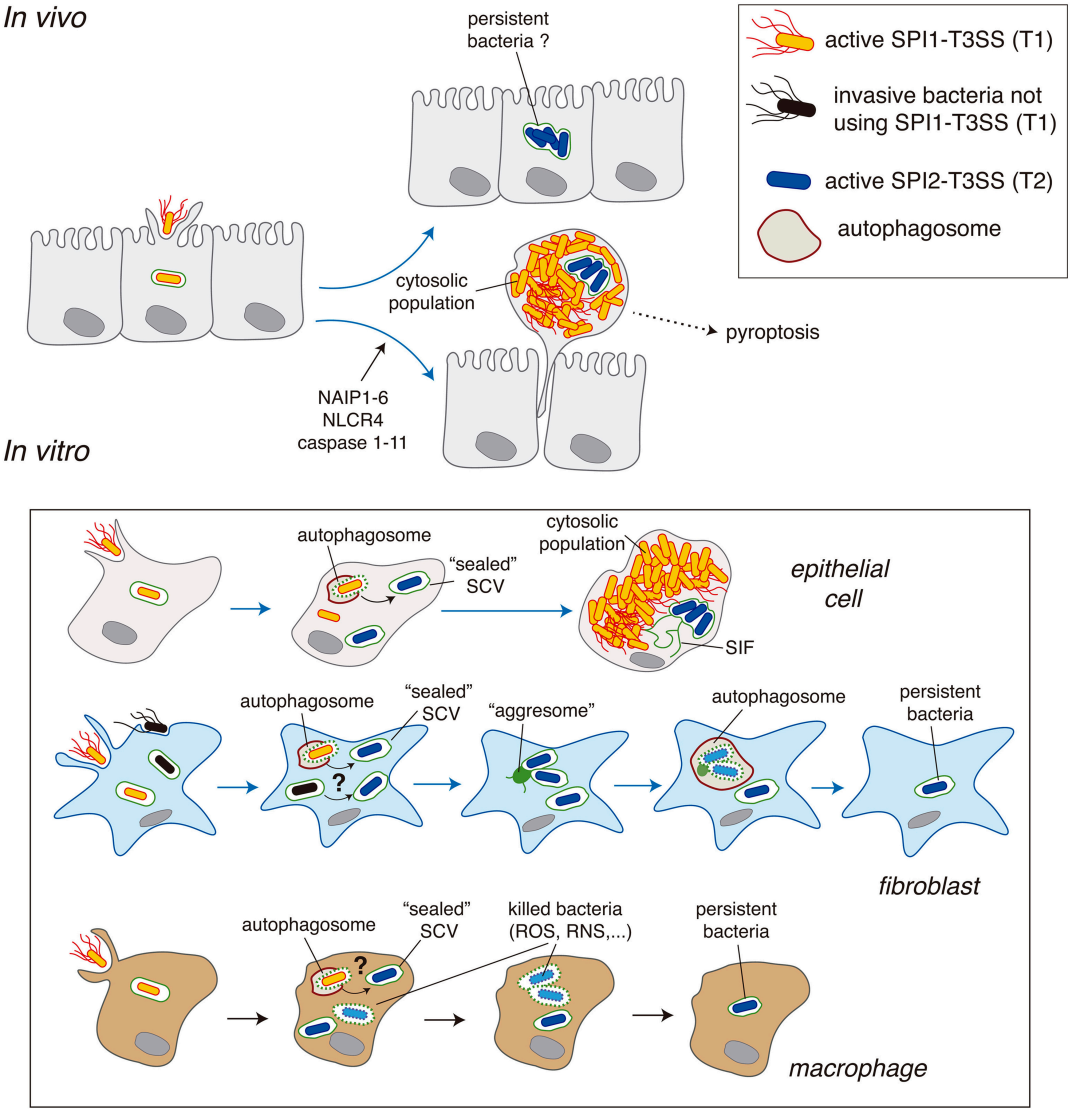
*Salmonella* intracellular populations reported in epithelial cells, fibroblasts and macrophages. A comparison of the data obtained *in vivo* and *in vitro* is also depicted. Abbreviations: NAIP1-6, neural apoptosis inhibitory proteins 1-6; NLRC4, NLR family CARD domain-containing protein 4; RNS, reactive nitrogen species; ROS, reactive oxygen species; SCV, *Salmonella*-containing vacuole; SIF, *Salmonella*-induced filaments; T1, SPI1-encoded type III secretion system; T2, SPI2-encoded type III secretion system.

To date, *Salmonella* intracellular subpopulations have been characterized mostly in epithelial cells, predominantly in the HeLa epithelial cell line derived from a human cervical cancer. In this cell line, ~10% of the initial intra-vacuolar population gains access to the cytosol, where bacteria proliferate at high rates to reach a progeny of >100 individuals per infected cell (Knodler, 2015). There is also *in vivo* evidence for hyper-replication of cytosolic *Salmonella* in the intestinal epithelium leading to a bacteria-induced extrusion of heavily infected enterocytes (Laughlin et al., 2014). *Salmonella* hyper-replication in the cytosol was also reported in the gallbladder epithelium and in polarized epithelial cells in which cell extrusion correlated with inflammatory cell death, pyroptosis, characterized by the release of IL-18 (Knodler et al., 2010). Bacteria-induced extrusion of epithelial cells is tightly regulated, with an active role of inflammasome components such as NAIP1-6, NLCR4 and caspase-1/-11 (Knodler et al., 2014a; Sellin et al., 2014; Figure 2). Heavily-infected cells are actively extruded in the gut lumen of wild type mice whereas bacterial loads in the lumen drop significantly in mice defective for inflammasome components (Sellin et al., 2014). This study also showed a large proportion of infected enterocytes (~90%) with a low average number (≤10) of intracellular bacteria. This population of infected enterocytes remained stable in numbers for the duration of the experiment (see Figure 1D in Sellin et al., 2014). Interestingly, these enterocytes mostly harbor intra-vacuolar bacteria (Sellin et al., 2014). Although these infected cells were not further examined by these authors, this intra-vacuolar population may act as a pathogen reservoir in the intestinal epithelium (Figure 2), an idea that could be tested in future studies. In conclusion, the cytosolic and intra-vacuolar *Salmonella* populations observed *in vitro* in cultured epithelial cells are also represented *in vivo*. Some few differences can however be noted. For example, *in vivo* the numbers of hyper-replicating cytosolic bacteria stay at an average of 30–40 per infected cell instead of the more than 100 bacteria per cell reported in most *in vitro* models (Knodler et al., 2014b).

*Salmonella* intracellular populations are less characterized in other eukaryotic cell types (Figure 2). As above mentioned, the cytosol of macrophages and fibroblasts are not permissive for proliferation of a *Salmonella sifA* mutant, which however hyper-replicates in the cytosol of epithelial cells (Beuzon et al., 2002). The *Salmonella* capacity to growth in the cytosol is therefore a host cell type-dependent trait. In agreement with these early observations, to date no study has reported hyper proliferation of cytosolic wild-type *Salmonella* either in macrophages or in fibroblasts. Despite early reports in mouse embryonic fibroblasts (MEFs) showing co-localization of 10–20% of intracellular bacteria with autophagy markers at early post-infection times (1–4 h) (Birmingham and Brumell, 2006), evidence for subsequent hyper-replication of this population was lacking. The restrictive nature of the fibroblast cytosol may involve: (i) lack of nutrient availability; (ii) an effective autophagy response, or, both factors that could prevent the generation of a stable cytosolic population. Similar conclusions could be reached in macrophages, in which evidence of a population of free actively growing cytosolic *Salmonella* is lacking despite TEM assays showing the eventual presence of some cytosolic bacteria in cultured macrophages (Perrin et al., 2004; Thurston et al., 2016b). To our knowledge, no study has provided evidence in macrophages of a stable population of “free” cytosolic bacteria at any post-infection time.

### Fibroblasts

Fibroblasts are ubiquitous non-phagocytic cells that play a key role in tissue homeostasis. Unlike in epithelial cells, there are no reports of *Salmonella* populations proliferating as free bacteria within these cells. In fibroblasts, a proportion of SCV may suffer damage early in the infection (1–2 h) as they recruit “danger” proteins that respond to glycans exposed in the inner (luminal) face of the SCV membrane. These include ubiquitin, LC3 or the autophagy adaptor protein CALCOCO2/NDP52 (Birmingham and Brumell, 2006; Lopez-Montero et al., 2016). Only recently, the dynamics of *Salmonella* populations in fibroblasts was deciphered in detail. Live-cell imaging microscopy of rat and human fibroblast cell lines uncovered a new form of autophagy taking place in response to *Salmonella* infection (Lopez-Montero et al., 2016). Whereas, ~10% of the SCV in these fibroblast cell lines are positive for CALCOCO2/NDP52 and ubiquitin at 2 hpi, at later infection times (>4 h) the *Salmonella* population is entirely intra-vacuolar showing no ubiquitin accessibility (Lopez-Montero et al., 2016). This intra-vacuolar population attempts to develop phagosomal extensions (SIFs) that further collapse into a static aggregate formed by host endomembranes (Lopez-Montero et al., 2016). This lack of stable SIFs agrees with the inability of intracellular *Salmonella* to translocate SifA in fibroblasts (Núñez-Hernández et al., 2014). Moreover, the reaction to this aggregate occurs via a non-canonical autophagy response that is independent of ubiquitin and CALCOCO2/NPD52. The autophagosome captures only part of the intra-vacuolar population, only those SCV in proximity to the aggresome (Lopez-Montero et al., 2016; Figure 2). These observations demonstrated the existence of two intra-vacuolar subpopulations, one having a lifespan of 8–12 h, time at which they are degraded inside the autophagosome and, a second intra-vacuolar population that persists in the infected cell for longer times. Live-cell imaging showed that this second intra-vacuolar population does not repeat the attempt to form SIFs, ensuring that no subsequent rounds of autophagy take place. This study illustrates how live-cell imaging microscopy can unravel phenomena related to the dynamics of defined *Salmonella* populations along the infection cycle. Live-cell imaging makes unnecessary the ectopic expression of key proteins involved in pathogen recognition or phagosome formation such as LC3 or p62 for tracing autophagy-related events (Birmingham and Brumell, 2006; Yu et al., 2014).

### Neutrophils

Neutrophils play a major role in controlling *Salmonella* infections (Keestra-Gounder et al., 2015; Crowley et al., 2016) and are more bactericidal than macrophages, mostly through the action of reactive oxygen species (ROS) (Miao et al., 2010). Despite this efficient killing activity, neutrophils recruited to the intestinal lumen in infections caused by non-typhoidal *Salmonella* do not eradicate all ingested bacteria (Loetscher et al., 2012). These luminal neutrophils have a limited life-span, which denotes a “transient” residence for the surviving bacteria. Whether cytosol and/or intra-vacuolar populations differentiate within these neutrophils is unknown.

## Methods to identify cytosolic and intra-vacuolar *Salmonella* populations

Intra-vacuolar and cytosolic *Salmonella* populations are distinguished by a variety of assays. We refer the reader to excellent recent reports that cover in depth these techniques (Knodler, 2015; Klein et al., 2017). Here, we briefly comment the bases and main features of the most commonly used assays. These include: resistance to chloroquine (CHQ); selective membrane permeabilization with digitonin; and, assessment by fluorescence microscopy of the distribution in infected cells of host membrane markers and autophagy proteins. These assays are often accompanied with estimates of the bacterial proliferation rate, calculated after determining the number of viable intracellular bacteria at distinct post-infection times.

The chloroquine resistance assay was used for the first time to quantify cytosolic *Salmonella* in a study conducted by Knodler and colleagues in multiple epithelial cell lines (Knodler et al., 2014b). This assay exploits the lysosomotrophic nature of CHQ, which accumulates in vacuolar acidic compartments (Knodler et al., 2014b; Klein et al., 2017). Due to this property, CHQ targets intra-vacuolar but not cytosolic bacteria and allows the selective quantification of cytosolic bacteria through enumeration of viable counts following exposure of infected cells to the drug.

An alternative method consists in the use of digitonin, a compound with detergent properties that permeabilizes more efficiently plasma membrane than endomembranes (Checroun et al., 2006; Meunier and Broz, 2015). Eukaryotic cells infected with a *Salmonella* strain expressing a fluorescent protein are differentially permeabilized with ditigonin at a concentration previously adjusted for not affecting endomembrane integrity. This adjustment is critical to validate the assay and requires prior tests with anti-*Salmonella* antibodies and antibodies recognizing a luminal region of lysosomal integral membrane glycoproteins such as Lamp-1/Lamp-2. A suitable digitonin concentration should prevent labeling of Lamp-1/Lamp-2, meaning that only the plasma membrane is permeabilized. The access of anti-*Salmonella* antibody to some, but not all, intracellular bacteria is inferred as co-existence of cytosolic and intra-vacuolar populations. The method therefore relies on a selective control of the vacuolar membrane integrity in cells exposed to digitonin.

**Figure 3:**
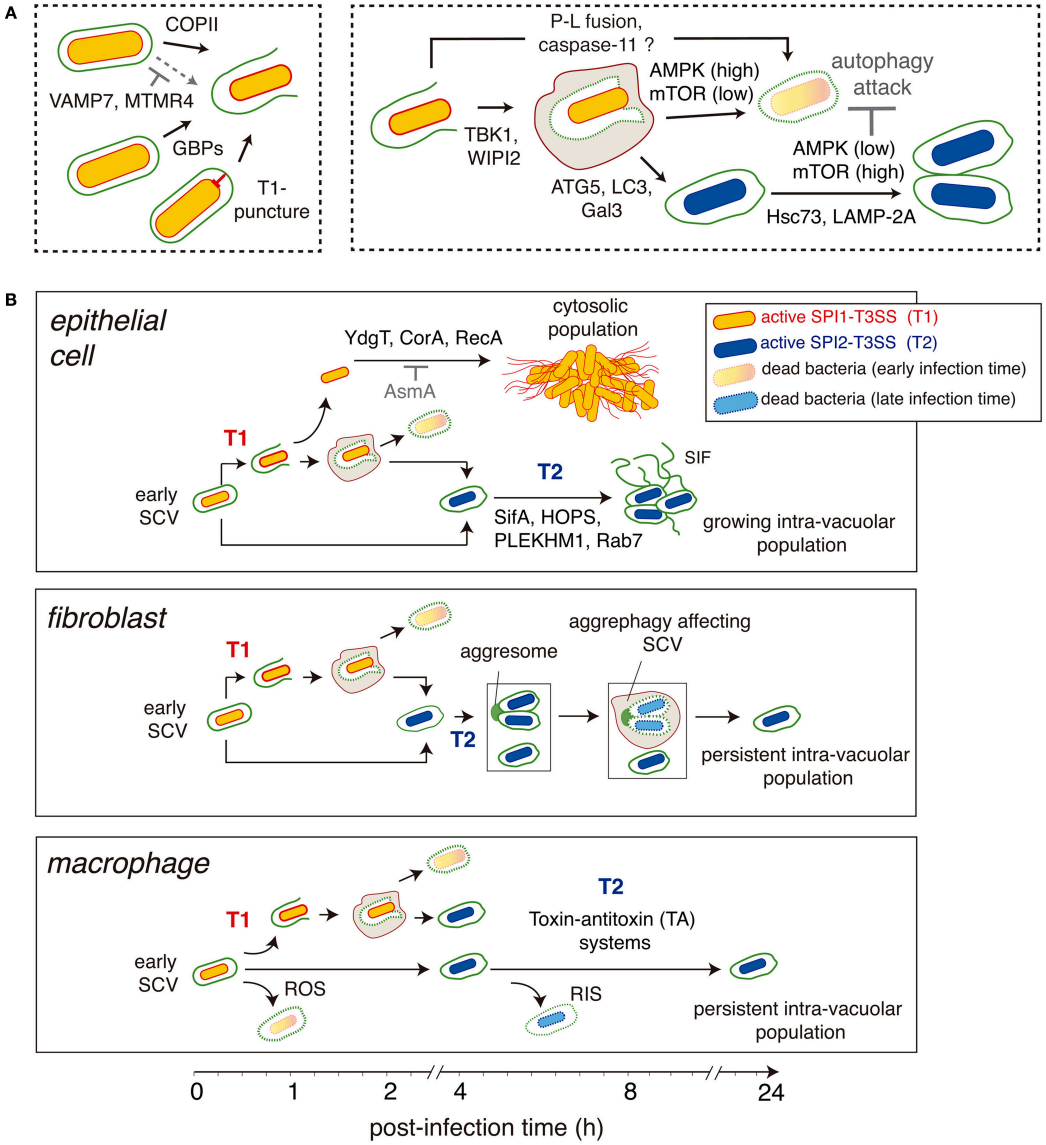
Different stages and main regulatory factors (host and pathogen origin) modulating the generation of the cytosolic and intra-vacuolar *Salmonella* populations. **(A)** Steps influencing the generation of cytosolic and intra-vacuolar populations. The factors involved are indicated. Those cases in which the effect is inhibitory are highlighted in gray; **(B)** Scheme depicting the main stages characterized in various host cell types (epithelial cell, fibroblasts, macrophages) regarding the generation of distinct bacterial populations as the infection progresses overtime. Factors known to contribute to defined steps are indicated. Abbreviations: AMPK, AMP-activated protein kinase; ATG5, autophagy protein 5; COPII, coat protein complex-2; Gal3, galectin-3; GBPs, guanylate-binding proteins; HOPS, homotypic fusion and vacuole sorting complex; Hsc73, heat shock cognate protein 73; LAMP-2A, receptor for chaperone-mediated autophagy; LC3, microtubule-associated proteins 1A/1B light chain 3B; MTMR4, myotubularin-4; mTOR, mammalian target of rapamycin; PLEKHM1, Pleckstrin homology domain-containing protein family member 1; P-L fusion, phagosome-lysosome fusion; RNS, reactive nitrogen species; ROS, reactive oxygen species; SCV, *Salmonella*-containing vacuole; SIF, *Salmonella-*induced filaments; T1, SPI1-encoded type III secretion system; T2, SPI2-encoded type III secretion system; VAMP7, vesicle membrane-associated protein 7; TBK1, Tank-binding kinase 1; WIPI2, WD repeat domain phosphoinositide-interacting protein 2.

To invade host cells, *Salmonella* uses the T3SS encoded in the *Salmonella* pathogenicity island 1 (SPI-1), hereinafter referred as SPI1-T3SS. Besides their role in promoting bacterial entry, T3SS of several pathogens involved in translocation of effector proteins are known to elicit damage in the phagosomal membrane (Hakansson et al., 1996; Roy et al., 2004; Birmingham et al., 2006; Kreibich et al., 2015). Interestingly, the damage caused by SPI1-T3SS in the SCV membrane can be repaired by the action of autophagy proteins like ATG5, Gal3 and LC3 (Kreibich et al., 2015; Figure 3A). These “repair” events must be considered when a cytosolic *Salmonella* subpopulation is suspected in the assay. Depending on the infection time, the selective digitonin permeabilization assay may classify as part of the cytosolic population some bacteria that are only transiently exposed to this compartment. This aspect also raises the question of whether intracellular bacteria surrounded by a damaged SCV membrane should be cataloged as “cytosolic” simply because antibodies can reach these bacteria in a digitonin assay. In these cases, probable co-localization of these bacteria with phagosomal membrane proteins should be examined. Stable transfectant cells expressing a fluorescent membrane marker protein that localizes in the SCV, e.g., Rab5, Rab7, or Lamp-1, are appropriate to monitor by time-lapse microscopy whether bacteria egress from the SCV into the cytosol to generate a subpopulation lacking membrane markers. Live-cell imaging microscopy, together with analysis of accessibility to compartments previously loaded with fluid endocytic marker like dextrans (Rajashekar et al., 2008; Malik-Kale et al., 2012), can be undoubtedly very informative.

The existence of a *Salmonella* cytosolic population in the assay can also be inferred by measurement of the pathogen intracellular replication rate. Cytosolic *Salmonella* hyper-replicate in epithelial cells with a peak of proliferation estimated at 8 h post infection for commonly used epithelial cell lines such as HeLa cells (Malik-Kale et al., 2012; Knodler et al., 2014b). At later post-infection times (~16 h), epithelial cells containing hyper-replicating *Salmonella* can detach and lose plasma membrane integrity allowing the entry of gentamicin, which can lead to underestimation of viable intracellular bacteria. This issue was recently addressed in a study focused in the contribution of SPI2-T3SS to replication of intra-vacuolar *Salmonella* (Malik-Kale et al., 2012). These authors showed that a defect in intra-vacuolar replication could be masked due to the population actively proliferating in the cytosol. TEM-based techniques, although labor intensive, can provide definitive support to the presence of “free” cytosolic bacteria (Beuzon et al., 2000; Knodler et al., 2010) or bacteria enclosed within membranes having compromised integrity (Laughlin et al., 2014).

Given the access of many labs to live-cell imaging and super-resolution microscopy, the field could certainly benefit from an increment in comparative studies involving distinct host cell types that are infected with the same bacterial strain and monitored overtime in parallel. This experimental design could precisely define when and how the distinct *Salmonella* intracellular populations emerge, stabilize or disappear. The benefits of this approach are well illustrated in the live-cell imaging study performed by Steele-Mortimer and colleagues in epithelial cells (Malik-Kale et al., 2012). These authors monitored the actively-growing cytosolic population in the presence/absence of a functional SPI2-T3SS to demonstrate the co-existence of the intra-vacuolar and cytosolic populations in some infected cells. New approaches based on fluorescence complementation with split-GFP allow to monitor overtime the distribution of *Salmonella* effectors in live infected cells (Young et al., 2017). This technique could be exploited to visualize effector distribution as the distinct intracellular populations emerge.

## Factors modulating phagosome-to-cytosol transition

### Pathogen factors

The egress of intra-vacuolar bacteria into the cytosol is only possible if loss of integrity in the phagosomal membrane occurs. Unlike many Gram-positive bacterial pathogens, Gram-negative pathogens are not armed with pore-forming toxins that alter and ultimately lyse the phagosomal membrane although they can produce deacylases and other types of lipases that disrupt the lipid bilayer (Ohlson et al., 2005; Linhartova et al., 2010).

Another common mechanism of membrane damage is that mediated by the insertion into the host eukaryotic membrane of the “translocon,” a protein complex that constitutes the most external part of T3SS apparatuses (Deng et al., 2017). The “puncture” of the phagosomal membrane by this complex can result in damage in the absence of any type-III effector (Du et al., 2016). This conclusion was reached when detecting free in the cytosol a recombinant *Escherichia coli* strain expressing the complete repertoire of *Shigella* type-III apparatus structural proteins but none of its effectors (Du et al., 2016). The *Shigella* translocon protein IpaC was responsible for the phagosomal escape of *E. coli* (Du et al., 2016). The *Salmonella* translocon ortholog protein SipC was slightly less efficient in promoting phagosomal lysis compared to IpaC, a phenotype observed in epithelial cells and macrophages. Interestingly, the *Yersinia* translocon proteins did not cause rupture of the phagosomal membrane (Du et al., 2016). Therefore, distinct translocon proteins of various T3SS affect the stability of the phagosomal membrane to different extents, which may have consequences for the fate of intracellular bacteria. Damage to the SCV membrane associated to the activity of the SPI1-T3SS has been corroborated by other studies (Brumell et al., 2002; Roy et al., 2004; Birmingham et al., 2006; Ibarra et al., 2010; Thurston et al., 2012).

Studies of the *Salmonella sifA* mutant, which displays increased release to the cytosol, showed that SCV membrane destabilization requires the SPI2-T3SS effector SseJ, with predicted deacetylase activity (Ruiz-Albert et al., 2002). SseJ then destabilizes the SCV when SifA is absent, a process favored in the presence of PipB2 and SopD2, two SPI2-T3SS effectors that recruit kinesin to the SCV. Accumulation of this cytoskeletal motor protein in the SCV vicinity may affect SCV membrane integrity (Dumont et al., 2010; Schroeder et al., 2010). This view of destabilizing factors that need to be counterbalanced by other proteins is a common theme that extends beyond *Salmonella* (Creasey and Isberg, 2014; Fredlund and Enninga, 2014). Thus, the integrity of the *Legionella*-containing vacuole depends on a delicate balance between two pathogen proteins, PlaA and SdhA (Creasey and Isberg, 2012).

### Host factors

Pathogen and host proteins can also act in a coordinated fashion to ensure integrity and correct maturation of the SCV membrane (Mcewan et al., 2015). Thus, SifA binds to the Pleckstrin homology domain-containing protein family member 1 (PLEKHM1) to ensure recruitment of a Rab7-PLEKHM1-HOPS complex to the SCV. This event facilitates fusion of detoxified lysosomes to the SCV (Mcewan et al., 2015; Figure 3B). This step is essential to allow expansion of the SCV membrane. Depletion of PLEKHM1 results in SCV with abnormal morphology, lack of phagosomal membrane extensions (SIF) and decreased pathogen proliferation. Intriguingly, the inhibitory effect on the pathogen linked to the lack of PLEKMH1 is more pronounced in fibroblasts and macrophages, cell types in which the intra-vacuolar population predominates (Figure 3B).

The TBK1 kinase was initially claimed to be a host factor required for SCV membrane integrity (Radtke et al., 2007). Loss of TBK1 function promoted access of bacteria to the cytosol, leading to increased bacterial loads. This function was shown to be conserved in fibroblasts, epithelial cells and macrophages (Radtke et al., 2007). More recent studies however assigned a slightly different function to TBK1 in relation to SCV membrane integrity. TBK1 is proposed to control *Salmonella* intracellular proliferation by targeting bacteria that are in “already” compromised SCV (Boyle et al., 2016; Figure 3A). TBK1 is tethered to these damaged SCV by the autophagy danger sensors optineurin/OPTN -bound to ubiquitin- or CALCOCO2/NDP52, this latter indirectly via the adaptors AZI2 or TBKBP1 (Boyle et al., 2016; Thurston et al., 2016a). TBK1 activation results in stimulation of WIPI2, a phosphoinositide-3-phosphate [PI(3)P]-binding protein that promotes autophagosome formation (Dooley et al., 2014; Thurston et al., 2016a). Thus, bacteria can be “transiently” exposed to the cytosol and subsequently confined in a closed compartment, the autophagosome, which prevent them from hyper-replicating in the cytosol. Of note, these studies were based on the use of *tbk1*^−/−^ knock-out MEF fibroblasts, in which distinct TBK1 constructs were ectopically expressed together with siRNA-mediated interference of distinct autophagy proteins (Thurston et al., 2016a). This work was based entirely on measurement of bacterial replication rates and, therefore, did not estimate the number of SCV undergoing membrane alteration and TBK1/WIPI2-mediated autophagosomal enclosure. Despite this, the proposed TBK1-WIPI2-dependent antibacterial mechanism (Boyle et al., 2016; Figure 3A) is consistent with observations in fibroblasts showing no SCV damage after 2 hpi (Lopez-Montero et al., 2016). Future studies should evaluate if the TBK1-WIPI2 network plays a similar role in epithelial cells or macrophages.

An important role in destabilization of the SCV in macrophages has been recently assigned to guanylate-binding proteins (GBP) (Meunier et al., 2014). GBP expression is enhanced following the induction of a type-I interferon response. These GBP proteins alter the SCV membrane integrity (Figure 3A), favoring the release of lipopolysaccharide (LPS) from bacteria located in the compromised SCV. This LPS further interacts with caspase and stimulates the non-canonical inflammasome pathway (Meunier et al., 2014; Crowley et al., 2017). In this model, GBP proteins act upstream of caspase-11 activation and are a requisite for SCV membrane alteration. Noteworthy, the exposure to the cytosol of glycan chains present in the luminal side of the SCV membrane favors autophagosome formation, returning the bacteria to a “sealed” phagosomal compartment and avoiding in this manner caspase-11 over-stimulation (Meunier et al., 2014). An aspect to be considered in this model is the evidence in macrophages of a *Salmonella* subpopulation of persistent intra-vacuolar bacteria that is generated due to the action of toxin-antitoxin systems (Helaine et al., 2014; Figure 3B). It would be of interest to analyze whether phagosomes harboring these persistent bacteria evade in macrophages recognition by the membrane-disrupting GBP proteins. Moreover, LPS can also be actively released from intra-vacuolar bacteria in the form of LPS-vesicles (García-del Portillo et al., 1997). Thus, disruption of the SCV membrane might not be absolutely required for exposing LPS to cytosolic caspase-11.

Other host factors such as the endoplasmic reticulum coat protein complex II (COPII) and VAMP7, involved in lysosomal vesicle trafficking, influence integrity of the SCV membrane as well (Figure 3A). Accumulation of COPII in the SCV membrane of epithelial cells is proposed to have a destabilization effect and, as consequence, facilitate bacterial egress into cytosol (Santos et al., 2015). Conversely, VAMP7-positive vesicles favor maturation of the SCV to the stage competent for SIF formation in the HeLa infection model (Santos et al., 2015). How the cell determines which SCV are directed to one of the other apparently antagonistic pathway is still unknown. Intriguingly, in the case of the *L. pneumophila* vacuole COPII is required for the generation of a replicative organelle and, therefore, plays a membrane stabilizing role (Creasey and Isberg, 2012).

A recent report also assigns to myotubularin-4 (MTMR4), a phosphoinositide 3-phosphatase, a stabilizing role for the SCV membrane (Teo et al., 2016). MTMR4 influences the amount of PI(3)P present in the SCV membrane and its absence leads to decreased bacterial loads, which correlates with enhanced co-colocalization of SCV with autophagy-related proteins such as LC3, galectin-8 and SQSTM1/p62 (Teo et al., 2016). These observations provide additional support to the important role that the PI content has in preservation of the SCV membrane integrity.

## Autophagy and the generation of distinct *Salmonella* intracellular populations

Exposure of *Salmonella* to cytosolic host defenses and the hyper-replication shown by the pathogen in this subcellular compartment of epithelial cells seem, at first glance, contrasting phenomena. One important aspect to consider is the co-localization of autophagy markers with damaged SCV or cytosolic bacteria, which occurs at early stages of the infection and then diminishes as the infection progresses. This was first noted by Brumell and colleagues for the LC3 autophagy marker in SCV harboring wild-type bacteria (Birmingham et al., 2006). Interestingly, the percentage of intracellular bacteria co-localizing with autophagy markers at late infection times is even lower in the case of the *sifA* mutant, consistent with its hyper-replication phenotype.

Why does autophagy seem to attempt to control *Salmonella* intracellular populations only early during the infection of host cells? Which are the mechanisms that elicit this host response and how are they subverted by intracellular *Salmonella*? Has autophagy evolved to destroy in all cases intracellular *Salmonella* or can it have beneficial effects ensuring persistence of intra-vacuolar bacteria?

An example of a beneficial effect is the role that two components of the host protein turnover pathway known as chaperone-mediated autophagy, Hsc73 and LAMP-2A, have in enhancing growth of intra-vacuolar *Salmonella*. These chaperones facilitate nutrient access to the pathogen from the cytosol to the SCV in both epithelial cells and macrophages (Singh et al., 2017). A recent study in epithelial cells supports the idea of autophagy acting only at early infection times, 1–2 hpi (Ganesan et al., 2017). This picture is different in fibroblasts, in which two autophagy “waves” were recently described: an early interaction with the pathogen as in other cell types (1–2 hpi) and a second response triggered at late times (≥6–8 hpi) by endomembranous aggregates connected to the SCV (Lopez-Montero et al., 2016; Figure 3B).

The available data provide insights into some of these questions and how autophagy may shape the generation of distinct *Salmonella* populations. A commonly accepted view is that autophagy responds to signals linked to the lack of integrity in the SCV membrane. These signals become active through the recognition of glycans present in the luminal side of the SCV and the ubiquitin decoration of the bacterial surface. The major objective of this initial attack is to enclose the invading bacteria in a specialized autophagosome capable of destroying the pathogen. As a general principle, any factor destabilizing SCV membrane integrity should favor autophagy attack while the contrary applies for the stabilization factors, regardless of whether they are of host or pathogen origin.

Using MEFs and epithelial cells, Hardt et al. showed that autophagy plays an important role in the balance of the intra-vacuolar and cytosolic *Salmonella* populations (Kreibich et al., 2015). Lack of a functional autophagy machinery results in a reduction in the proportion of intra-vacuolar bacteria, with more bacteria escaping and hyper-replicating in the cytosol. This phenotype was further linked to a capacity of the autophagy machinery to “seal” damaged SCV. Thus, in the absence of a functional SPI1-T3SS, responsible for destabilizing the SCV membrane (Tattoli et al., 2012a), the proportion of intra-vacuolar bacteria remains constant regardless of the functionality of the autophagy machinery. An issue overlooked in this study is the capacity of *Salmonella* to invade fibroblasts using a SPI1-independent “zipper” entry route (Aiastui et al., 2010; Velge et al., 2012; Boumart et al., 2014). Such invasion route should not provoke SCV membrane damage and, therefore, not recruit autophagy. The SPI1-independent pathway can be responsible in fibroblasts for invasion rates of up to 20% of that registered for the SPI1-mediated route (Aiastui et al., 2010). A follow-up of a SPI1-defective *Salmonella* strain in fibroblasts regarding its capacity to proliferate or its distribution in vacuolar or cytosolic populations could add valuable insights into this issue.

Another important regulator of autophagy involved in modulating *Salmonella* populations is the mammalian target of rapamycin –mTOR-, a metabolic regulator-. This protein is a serine/threonine kinase that nucleates two distinct protein complexes known as mTOR-complex-1 (mTORC1) and mTORC2. Various processes including cell survival, motility, transcription and autophagy are regulated by mTOR (Saxton and Sabatini, 2017). Several studies have linked mTOR activity with *Salmonella* infection and autophagy induction (Tattoli et al., 2012a,b; Ganesan et al., 2017). These reports agree in a model of autophagy acting on *Salmonella* exposed to the cytosol early in the infection, coincident with a drop in mTOR activity (Figure 3A). Such alteration in mTOR has been proposed in epithelial cells to be associated to an early stage of amino acid starvation in *Salmonella*-infected cells, a stress response that leads to mTOR inactivation and induction of autophagy (Tattoli et al., 2012b). Interestingly, mTOR is reactivated at later times concomitantly to amino acid uptake and increased co-localization of mTOR with the SCV, which ensures autophagy escape (Figure 3A). Although this study dissected in detail the mechanisms of autophagy induction and further inhibition in the course of *Salmonella*-infection, there was no comparison regarding mTOR activity and the progression of the intra-vacuolar and cytosolic populations, which are easily differentiated in HeLa cells. A more recent study performed in macrophages has provided new insights into how mTOR could reactivate in *Salmonella*-infected cells (Ganesan et al., 2017). These authors show that AMPK, a kinase that becomes active when the energy status -ATP levels- drops, is targeted for degradation in lysosomes by intracellular *Salmonella* together with two of its positive regulators, LKB1 and Sirt. AMPK acts directly as a negative regulator of mTOR, so lowering activity of AMPK automatically results in increased mTOR function and decreased autophagy (Figure 3A). Of interest, this study shows that targeting of the LKB1/Sirt1/AMPK checkpoint depends on a functional SPI2-T3SS (Ganesan et al., 2017), a feature of the intra-vacuolar population (Knodler et al., 2010, 2014b). Similar to the study of mTOR in epithelial cells, the dynamics of *Salmonella* populations in macrophages having an active/inactive LKB1/Sirt1/AMPK circuit was not examined.

Although the mechanism is still unknown, the aggrephagy uncovered by live-cell imaging microscopy in *Salmonella*-infected fibroblasts has two unique features not described in any other cell type (Lopez-Montero et al., 2016). First, the autophagosome forms only at late infection times (from 6 to 8 h) and degrades its cargo slowly for periods that last up 16 h post-infection (see Figure 1C in Lopez-Montero et al., 2016). Second, these “late autophagosomes” are negative for autophagy markers such ubiquitin and CALCOCO2/NDP52. This evidence implies the ingestion and degradation of “intact” SCV, leaving untouched a population of persistent intra-vacuolar bacteria (Figure 3B).

## Concluding remarks

The evidence accumulated to date supports the existence of distinct *Salmonella* intracellular populations, some of them with short half-lives as it is the case of bacteria enclosed within SCV having damaged membranes. This subpopulation can be then considered a transient intermediate between the initial intact SCV and bacteria replicating free in the cytosol. The recent literature is providing examples of elaborated mechanisms by which the host and the pathogen dispute for shifting the equilibrium toward the intra-vacuolar and cytosolic population. In this line of reasoning, one should however be cautious considering that the pathogen may, under defined circumstances, prefer staying within a closed vacuolar compartment instead of hyper-replicating in the cytosol. The elegant studies of Steele-Mortimer, Knodler et al. favor the idea of cytosolic replication as a trait that may have integrated during *Salmonella* evolution to trigger the extrusion of the heavily-infected epithelial cell and to load the lumen with large bacterial numbers (Knodler et al., 2010; Sellin et al., 2014). The existence of *Salmonella* factors that regulate positively or negatively the growth rate in the cytosol of epithelial cells such as YdgT, CorA, RecA, or AsmA (Wrande et al., 2016), favors the notion of a specific program involved in the differentiation of the cytosolic population. Recent data also supports the idea of cytosolic bacteria targeting the pro-survival kinase Akt to prolong the lifespan of the infected cell (Finn et al., 2017). This cytosolic population stimulating cell extrusion from the epithelium contrasts to that of the infected epithelial cells remaining in the epithelium and harboring low numbers of bacteria that are mostly intra-vacuolar (Sellin et al., 2014). This bacterial population may act as a potential persistent reservoir that should be analyzed in detail in future studies.

Despite the evidence found for the *Salmonella* cytosolic lifestyle, the intra-vacuolar lifestyle is, in our opinion, predominant for defining the biology of *Salmonella* inside eukaryotic cells. A recent study by Hensel et al. provides proof for the nutritional function of the intricate endomembrane network of which SIF forms part, which probably evolved to facilitate nutrient acquisition to the intra-vacuolar bacterial population (Liss et al., 2017). Moreover, a plethora of *Salmonella* T3SS effectors have evolved to work in concert to ensure correct biogenesis, subcellular location and maintenance of the SCV (Deng et al., 2017), which must have an evolutionary basis. The evidence collected to date regarding persistence of *Salmonella* inside the infected cell also point to a privileged vacuolar compartment to do so, either in macrophages (Helaine et al., 2014) or other host cell types as fibroblasts (Núñez-Hernández et al., 2013).

We should also consider the power of techniques such as live-cell (time-lapse) imaging microscopy and single cell analyses to monitor the evolution of *Salmonella* intracellular populations, i.e., how they develop and how they are favored or counter-selected along the infection. The bulk of high throughput data obtained in varied infection models should facilitate the use of computational tools to raise new predictive models valuable to examine intracellular population dynamics and the heterogeneity in pathogen and host responses, which are currently studied intensively (García-del Portillo, 2008; Sanchez-Romero and Casadesus, 2014; Bumann, 2015; Saliba et al., 2016; Rego et al., 2017). Host responses can be differentiated not only in subpopulations of infected cells harboring either reduced or high bacterial loads (Saliba et al., 2016) but also in bystander non-infected cells (Ramos-Marquès et al., 2016). The generation of subpopulations in the host and the pathogen progeny are therefore intimately-related phenomena. Unravelling this communication will be a major goal for future studies, especially regarding the signals that program to be part of one or another population.

## Author contributions

FG reviewed the literature and wrote the manuscript; SC reviewed the literature, discussed data and provided editorial input.

### Conflict of interest statement

The authors declare that the research was conducted in the absence of any commercial or financial relationships that could be construed as a potential conflict of interest.
